# Paroxysmal hyperthermia, dysautonomia and rhabdomyolysis in a patient with Lesch–Nyhan syndrome

**DOI:** 10.1002/jmd2.12249

**Published:** 2021-09-28

**Authors:** Mandeep Rana, Karen Cuttin, Gerard T. Berry, Alcy Torres

**Affiliations:** ^1^ Department of Pediatrics, Division of Pediatric Neurology Boston University School of Medicine, Boston Medical Center Boston Massachusetts USA; ^2^ Department of Pediatrics, Division of Genetics and Genomics Boston Children's Hospital and Harvard Medical School Boston Massachusetts USA

**Keywords:** dysautonomia, Lesch–Nyhan syndrome, neuroleptic malignant syndrome, rhabdomyolysis

## Abstract

Lesch–Nyhan syndrome is an x‐linked genetic disorder of purine metabolism that results in the overproduction of uric acid and neurologic deficits manifesting as intellectual disability, dystonia, other movement disorders and self‐mutilation. We describe a 12‐year‐old patient with a history of Lesch–Nyhan syndrome, G6PD deficiency and central diabetes insipidus and multiple admissions for fever, acute kidney injury and transaminitis in the setting of rhabdomyolysis. The patient's temperature dysregulation and dysautonomia is likely attributable to abnormal neurotransmitter release, particularly that of dopamine, in the central nervous system. Our patient presented similarly to that of a patient with neuroleptic malignant syndrome (NMS), with symptoms including altered mental status, fever, dysautonomia and renal failure, and laboratory findings including elevated serum creatinine kinase, leukocytosis, transaminitis, hypernatremia and metabolic acidosis. Similar to NMS, disruption of dopamine neurotransmission results in dysregulated sympathetic activity and hyperthermia.

1

**Key Points**

‐ Lesch–Nyhan syndrome is an x‐linked genetic disorder of purine metabolism that results in the overproduction of uric acid and neurologic deficits manifesting as intellectual disability, dystonia, other movement disorders, and self‐mutilation.

‐ Twelve‐year‐old patient with a history of Lesch–Nyhan syndrome, G6PD deficiency, and central diabetes insipidus, and multiple admissions for fever, acute kidney injury, and transaminitis in the setting of rhabdomyolysis.

‐ The patient's temperature dysregulation and dysautonomia are likely attributable to abnormal neurotransmitter release, particularly that of dopamine, in the central nervous system.

‐ Similar to neuroleptic malignant syndrome (NMS), disruption of dopamine neurotransmission results in dysregulated sympathetic activity and hyperthermia.

## 2 INTRODUCTION

Lesch–Nyhan syndrome is an x‐linked genetic disorder of purine metabolism due to hypoxanthine‐guanine phosphoribosyltransferase (HGPRT) deficiency, which results in hyperuricemia. This syndrome is characterised by an array of neurological dysfunctions that include intellectual disability, dystonia, choreoathetosis, spasticity and compulsive uncontrollable self‐mutilation that in part originate from striatal dysfunctions.

Episodes of hyperthermia identical to those of neuroleptic malignant syndrome (NMS) have been reported in Lesch–Nyhan syndrome in the absence and presence of a neuroleptic agent.^1^ The literature attributes this phenomenon to an autonomic imbalance resulting from abnormal neurotransmitter release in the extrapyramidal system.^1^, ^2^, ^3^, ^4^ Disruption of dopamine neurotransmission leads to dysregulation of efferent sympathetic activity.^5^, ^6^, ^7^

NMS is a life‐threatening neurologic emergency characterised by altered mental status, muscle rigidity, fever and dysautonomia. Associated lab findings include elevated serum creatinine kinase (over 1000 IU/L; leukocytosis; transaminitis; elevated lactate dehydrogenase and alkaline phosphatase levels; electrolyte abnormalities, such as hypernatremia and metabolic acidosis; acute renal failure; and iron deficiency.^8^, ^9^, ^10^, ^11^ Antipsychotic and antiemetic agents are typically associated with NMS. Associated risk factors for NMS include withdrawal from antiparkinson medications, psychiatric conditions, acute catatonia and extreme agitation. Dehydration, which is present in 92% of patients, may be a risk factor or sequelae of NMS.

We report a patient with Lesch–Nhyan syndrome who has presented with multiple episodes of hyperthermia and metabolic derangements resembling that of NMS.

## 3 CASE REPORT

Our patient is a 12‐year‐old male with Lesch–Nyhan syndrome in addition to a G6PD deficiency, central diabetes insipidus (DI), new‐onset epilepsy, congenital microcephaly and failure to thrive with gastrostomy dependence. He presented to a Massachusetts Emergency Department (ED) with a fever of 103 °F, labile blood pressure, altered mental status, worsening irritability and poor sleep, non‐bilious and non‐bloody emesis and several episodes of non‐bloody diarrhoea.

Our patient was diagnosed with Lesch–Nyhan syndrome at approximately 4 years of age. Sequence analysis and deletion/duplication testing demonstrated sequence change c.191C > A (p.Ala64Asp) on Exon 3 of HPRT1 gene. He had global developmental delay and microcephaly and is now wheel‐chair dependent. Currently, he has limited language ability but can understand simple commands and many single common words. Intermittent dystonic posturing, self‐injurious and mutilating behaviours began at age 15 months and have worsened over the years. His parents have not been willing to allow teeth extraction. He was diagnosed with central DI by endocrinologist at age 10 years when he presented with hypernatremia. His serum sodium was between 158 and 160 mmol/L (135–145 mmol/L) that was refractory to free water replacement. Urinary Na was <40 meq/L, plasma osmolality was 376 months/kg (275–295 months/kg H2O), and urinary osmolality was 605 (50–1200 months/kg H2O). These abnormalities resolved following desmopressin administration.

During this current presentation on examination, he exhibited both pyramidal and extrapyramidal signs including spasticity in the upper and lower extremities, severe generalised dystonia, choreoathetosis and ballismus. His cranial imaging has been normal with the exception of lack of posterior pituitary signal, which can be seen in setting of DI.

Over the years, he has been tried on several medications to help with tone abnormalities, compulsive behaviours and disturbed sleep patterns. The patient's home medication regimen prior to hospitalisation included desmopressin 0.5 mg daily for central DI, levetiracetam 500 mg twice daily for seizures, clonazepam and tetrabenazine 25 mg twice daily for movement disorder. He is also on clonidine 0.4 mg daily for agitation, allopurinol for hyperuricemia and supplemental vitamin D. Addition of tetrabenazine improved dystonia by more than 50% per mother's report.

Two days prior to presenting to the ED, our patient began experiencing increased agitation and poor sleep. The patient did not experience additional infectious symptoms or seizure activity, denied a history of trauma or ingestion, and was compliant with medications.

In the ED, the patient appeared fatigued and lethargic, and his exam was notable for severe generalised dystonia. He had a fever of 103 °F, tachycardia reaching 150 BPM, tachypnea in the 30s and hypotension with systolic levels dropping to 80 mmHg from normal at times. The patient, however, had sufficient oxygen saturation and did not demonstrate laborious breathing. Metabolic studies were significant for creatinine (5.19 mg/dl), sodium (159 mmol/L), potassium (4.4 mmol/L) and chloride (121 mmol/L); transaminitis was noted with AST and ALT levels of 396 and 141 U/L, respectively. Complete blood count was significant for leukocytosis, with a white cell count of 23.1 K/μl and 84% polymorphonuclear leukocytes. Lactate was mildly elevated (2.2 mmol/L). Urinalysis was reassuring against infection. Creatinine kinase was elevated (29 000 U/L). Venous blood gas revealed a pH of 7.31, a PaCO2 level of 31 mmHg and a bicarbonate value of 15.6 mmol/L, suggesting anion gap metabolic acidosis with respiratory compensation. Although an infectious work‐up in the ED was negative (respiratory viral panel, chest x‐ray and urinalysis were within normal limits), the patient was empirically treated with ceftriaxone while awaiting blood and urine cultures.

The patient's presentation was concerning for hypovolemia, hypernatremic shock, acute kidney injury and rhabdomyolysis of unclear aetiology. He was admitted to the paediatric intensive care unit (PICU) for fluid resuscitation and management of rhabdomyolysis. During his PICU stay, he received hyperhydration with intravenous fluids. Tetrabenazine was stopped for benefit of doubt. Dopa agonists were not used. His urine output, creatinine kinase, electrolytes and transaminase levels were carefully monitored. Creatine kinase and aspartate aminotransferase peaked at 58 223 and 1203 U/L, respectively, before normalising. His electrolyte imbalance and renal function gradually improved to normal. On musculoskeletal exam, the patient had tenderness upon palpation of the left hip, localised erythema and effusion and pressure ulcers, increasing his risk of osteomyelitis and bacteremia. Consequently, hip x‐ray and joint arthropathy were performed, and cefazoline was empirically started to rule‐out septic arthritis. Although initially febrile during the hospital stay, the patient's infectious workup, including arthropathy and blood and urine cultures, was negative, and his episodes of diarrhoea and fever resolved. Renal and liver ultrasounds reassured against urinary obstruction and liver damage, respectively. When the patient became hemodynamically stable and the rhabdomyolysis and acute kidney injury resolved, he was transferred to the general paediatrics ward for ongoing monitoring during advancement of enteral feeds before being discharged.

It is worth noting that the patient started having intermittent fevers ranging from 100.4° to 103 °F since the age of 7 years, independent of associated symptoms, mental status changes, identifiable infectious etiologies or negative infectious workups. These would last between 1 and 2 weeks and self‐resolve. He was not on any dopaminergic medications at this time and not had prior admissions for similar presentations. The most recent hospital admission had occurred within the last 2 years. He also had at least four similar presentations necessitating admission to the ICU, one of which was in the absence of a dopaminergic antagonist.

Since discharge, the patient has been diagnosed with paroxysmal hyperthermia with associated metabolic abnormalities, including hypernatremia and rhabdomyolysis, presumed to be central in origin.

## 4 DISCUSSION

In a case report by Nyhan and Lucas, similar reports of two patients with Lesch–Nyhan syndrome who experienced episodes of hyperthermia comparable to NMS in the absence of neuroleptic agents are described.^1^ One of the cases describes a patient who experienced frequent episodes of fevers of up to 107.2 °F, leukocytosis with left shift, a negative infectious workup, metabolic acidosis, rhabdomyolysis with creatinine kinase of up to 75 000 μ/ml, somnolence and ileus; this clinical presentation strongly mirrors that of our patient.^1^ The patient began experiencing these syndromes at 7 years of age, and the frequency of events increased drastically, such that home management was initiated, requiring the use of a cooling blanket, rehydration therapy and medical management.^1^ It is worth noting that this patient occasionally received treatments with neuroleptic agents, such as aripiprazole, but had several syndromes independent of medications.^1^ The other case discussed in the report by Nyhan and Lucas involves a deceased patient in his twenties with various episodes of fever of unknown origin beginning in his mid‐teenage years.^1^ Our patient began having paroxysmal hyperthermia at 7 years of age, indicating that this phenomenon manifests at different ages; however, the similarities in the sequelae reported in these cases are remarkable and suggest a strong underlying association with Lesch–Nyhan syndrome.

One caveat that was considered in our patient's case was the potential role of tetrabenazine (TBZ) in the manifestation of his symptoms. TBZ is used to manage movement disorders involving chorea, dystonia, tardive dyskinesia and Tourette syndrome. TBZ inhibits the uptake of monoamines, particularly that of dopamine, thereby gradually depleting stores and possibly leading to NMS; however, our medical team hypothesized that this was not the aetiology of our patient's paroxysmal hyperthermia and associated metabolic abnormalities given the sudden and intermittent nature of his episodes. In between events of hyperthermia, our patient is asymptomatic and without electrolyte derangements. Moreover, our patient's first episode of fever of unknown origin began in 2011, at which point our patient was not yet being treated with tetrabenazine. However, tetrabenazine was discontinued during the admission.

Our patient instead received a diagnosis of paroxysmal hyperthermia with associated metabolic abnormalities, including hypernatremia and rhabdomyolysis, presumed to be central in origin in the setting of Lesch–Nyhan syndrome. This syndrome has been associated with autonomic imbalances owing to dysregulated neurotransmission and sympathetic activity in the extrapyramidal system.^1^, ^2^

In a study conducted on brain regions of post‐mortem patients with Lesch–Nyhan syndrome, alterations were found in levels of various neurotransmitters in the central nervous system (CNS).^2^ Most biochemical indices of neurologic function in dopamine‐neuron terminals in the striatum were decreased up to 30% compared with controls; moreover, loss of nigrostriatal and mesolimbic dopamine terminals was reported.^2^

Striatal cholinergic function was also decreased, while GABA and norepinephrine levels were normal, and serotonin‐neuron terminal function was increased.^2^ While disturbances in neurotransmitters in the extrapyramidal system have been associated with some of the movement disorders found in Lesch–Nyhan syndrome, we propose that such dysregulation in the CNS may also provoke episodes of paroxysmal hyperthermia and metabolic derangements that manifest like NMS.^3^, ^4^

The pathogenesis of NMS has been attributed to the dopamine receptor blockade in the striatum and hypothalamus as inducers of thermogenesis and reduced heat dissipation.^5^, ^6^ Additionally, the dysregulation of dopamine neurotransmission can provoke destabilisation of sympathetic activity, manifesting as increased muscle metabolism, hypertonia, dysregulated vasomotor activity and a blunted vasopressor response.^5^, ^6^, ^7^

This hyperthermia rhabdomyolysis episode can be seen in other neurotransmitter imbalances, such as cholinergic surge or hyperserotonergic state. A potential mechanism might be the presence of a relative hypercholinergic state, due to low dopaminergic neurotransmission and loss of balance between dopamine‐acetylcholine systems, which might help differentiate these episodes.

Other consideration is that a possible causal link among hypernatremia, hyperosmolality and rhabdomyolysis has been provided by reported cases in central DI, in the absence of potential causes.^8^, ^9^, ^10^ The proposed mechanism is that electrolyte abnormalities lead to cell membrane disruption because of deranged sodium‐potassium‐ATPase pump function.^11^

This case adds a novel presentation of Lesch–Nyhan syndrome involving paroxysmal hyperthermia and metabolic abnormalities and proposes fluctuations in dopamine in the extrapyramidal system and subsequent dysregulation of the sympathetic nervous system as mechanisms for pathogenesis.

## 5 CONCLUSION

In this report, we presented a 12‐year‐old patient with a history of Lesch–Nyhan syndrome, G6PD deficiency and central DI who had multiple admissions for fever, acute kidney injury and transaminitis in the setting of rhabdomyolysis. Our patient's clinical picture resembled that of NMS, with symptoms including altered mental status, fever, dysautonomia and renal failure, and laboratory findings, including elevated serum creatinine kinase, leukocytosis, transaminitis, hypernatremia and metabolic acidosis. We propose that our patient's paroxysmal temperature dysregulation, dysautonomia and associated metabolic abnormalities are manifestations of Lesch–Nyhan syndrome, as similar cases have been presented in the literature. We attribute the pathogenesis of this phenomenon to be central in origin, as alterations in CNS neurotransmitter release, particularly dopamine, result in dysregulated sympathetic activity and hyperthermia.

However, central DI deserves consideration. If it has contributory role or is the sole aetiology of this paroxysmal presentation in a child with Lesch–Nyhan syndrome remains a question for further inquiry.

## CONFLICT OF INTEREST

The author declares that there is no conflict of interest that could be perceived as prejudicing the impartiality of the research reported.

## Data Availability

My manuscript has no associated data.
